# Trace2PS and FSA2PS: two software toolkits for converting trace and fsa files to PostScript format

**DOI:** 10.1186/1751-0473-4-4

**Published:** 2009-07-21

**Authors:** Justina Krawczyk, Alexander Goesmann, Ralf Nolte, Martin Werber, Bernd Weisshaar

**Affiliations:** 1 Computational Genomics, Center for Biotechnology (CeBiTec), 33594 Bielefeld, Germany; 2 Bioinformatics Research Facility (BRF), Center for Biotechnology (CeBiTec), 33594 Bielefeld, Germany; 3 Department of Developmental Genetics, Max-Planck-Institute for Molecular Genetics, 14195 Berlin, Germany; 4 Institute for Medical Genetics-CBF, Charité-University Medicine Berlin, 12203 Berlin, Germany; 5 Chair of Genome Research, Bielefeld University, 33594 Bielefeld, Germany

## Abstract

**Background:**

Due to the advanced techniques in sequencing and fragment analysis, DNA sequencers and analyzers produce vast amounts of data within short time. To administrate the large data volume conveniently, efficient data management systems are used in order to process and to store sequencers' or analyzers' data outcome. The inclusion of graphical reports in such systems is necessary to achieve a comprehensive view of the integrated data. However, the resulting data of sequencing and fragment analysis runs are stored in a proprietary format, the so-called trace or fsa format, which is only readable by programs provided by the instrument's vendor operating on the machine itself or by commercial tools designed for editing the respective data. To allow for a quick conversion of the proprietary data format into a commonly used one, toolkits are required that reach this aim and can be easily integrated into workflow systems.

**Results:**

We have developed the software toolkits Trace2PS and Fsa2PS which allow to convert sequence and fragment analysis raw data files to PostScript images, respectively. The toolkits are implemented as Perl modules that can be used as standalone command line applications in conjunction with a script-based analysis pipeline, or integrated in software applications for displaying the data content of trace and fsa files. The converter modules support the commonly used file formats for storage of sequencing (ABI and SCF) and fragment analysis data (FSA).

**Conclusion:**

The software toolkits provide useful applications to convert sequencing and fragment analysis files from a proprietary into a more common, human-readable format. Trace2PS and FSA2PS are useful and capable in data management workflow systems like SAMS, or laboratory information systems that are used for displaying trace and fragment analysis results via web-based tools over an intranet or internet connection to users that can view their results directly on the screen.

## Background

DNA sequencing techniques have emerged to fast and customized analysis methods. Nowadays, DNA sequencers permit cheap large-scale genome sequencing producing vast amounts of data within a very short time [1]. Besides DNA sequencing, DNA fragments analysis is another indispensable high-throughput technology for basic research in molecular biology laboratories. Fragment analysis is used for genotyping and to investigate genetic diversity as done by Woodhead *et al*. [2] to reveal genetic differences between *Arabidopsis lyrata *and *Arabidopsis thaliana *by microsatellite development. Current analysis instruments like the Applied Biosystems 3730 × l DNA Analyzer can run up to 96 fragment analysis samples in parallel in a period of a few hours.

Because of the huge amount of data produced by the present generation of sequencers and analyzers, the resulting data is processed and stored using efficient data management systems, even if single reads or fragment analysis are to be studied. Graphical reports included in data management systems are very useful to access the results, and to get a quick overview of the data. However, sequencers and analyzers usually produce data in proprietary formats, which are only readable by programs provided by the instrument's vendor operating on the machine itself or by commercial tools designed for editing the respective files. The most commonly used formats for storing Sanger-type DNA sequencing data are ABI and SCF [3]. Sequencing files are referred to as traces and encompass the trace amplitudes, base calls and their confidence values, as well as textual data of the particular sequencing experiment. For storing fragment analysis results, a common file format is FSA that has been introduced by Applied Biosystems [4]. There are several tools required to convert sequence traces and fragment analysis result files into a more common, human-readable file format. At the same time, this transformation should be capable of easily being integrated into workflow systems that are used to process and manage DNA analysis data. A very portable format for displaying sequencing and fragment analysis results is PostScript that can be quickly converted into other commonly used formats like PDF. To reach the goals mentioned above, we have developed two toolkits; one for converting common sequencer file formats and the other one for converting fragment analysis formats to PostScript.

## Implementation

The two software toolkits are implemented as Perl modules which convert trace and fsa files into PostScript format: Trace2PS and Fsa2PS. The toolkits consist each of a module and an application script written in Perl that provides a front-end, and exemplifies the utilization of the corresponding module. The Perl modules are packaged in a CPAN [5] compatible archiving format referred to as CPAN distribution. Hence, the modules can be easily integrated by an installation script, commonly included in CPAN distributions, into the user's system.

## Results

### Trace2PS

**Figure 1:**
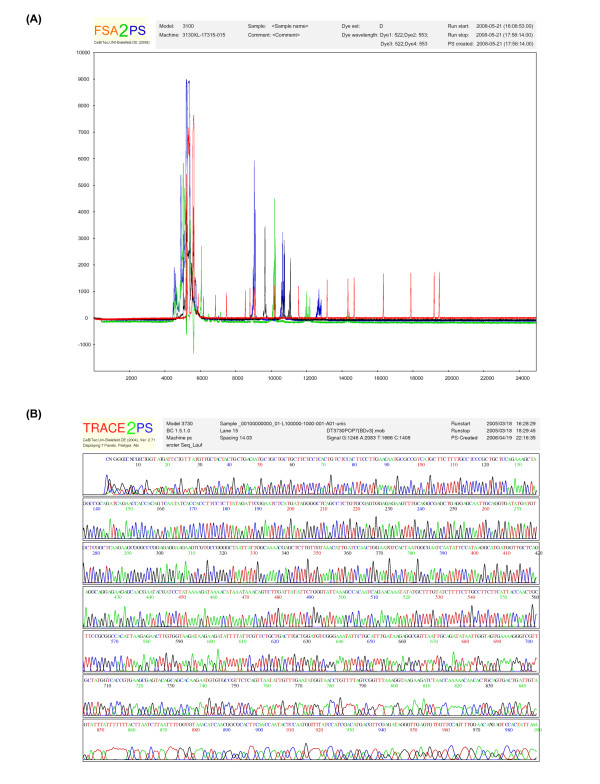
**PostScript output of Fsa2PS (A) and Trace2PS (B). **PostScript output of Fsa2PS (A) and Trace2PS (B). The header fields comprise each selected information on the sequencing or fragment analysis run, respectively. (A) The result of a typical fragment analysis run is presented. Here, microsatellites of an example organism were investigated.  (B) By default, the sequencing result is subdivided into seven panels.  Above the chromatogram, the derived base sequence is shown.

Currently, Trace2PS supports the widely used trace file formats ABI and SCF. The file format is detected automatically while the file is passed from command line. As a default, Trace2PS will output a PostScript file containing the trace display together with a plain text file containing the DNA sequence represented by the respective trace, which is specified by the file extension .seq. Figure 1(B) shows a typical PostScript output of Trace2PS. By default, the trace view is segmented into seven panels each including a constant number of sequence reads. The number of panels to display can be changed optionally, but by decreasing this number less data will be shown within the output. The header field contains a selection of the most important information on a trace such as sequencer model name, base caller version, machine, sample, lane number, spacing, dye set, signal, run start and stop time as well as the creation time of the PostScript. Especially for SCF format, the content of the information field can be adjusted by user-defined values passed as command line options. Thus, the user can supplement the field content by missing trace file items or customize the content by overwriting the default.

### FSA2PS

With the emerging need for converting FSA files to PostScript, we have also developed the toolkit Fsa2PS for that we have adopted and adjusted all PostScript generating functions from Trace2PS. Fsa2PS utilizes the Perl extension Bio::Trace::ABIF available from CPAN for parsing the content of FSA files. To maintain the colour coding as used by the analyzer software for presenting genotyping results on screen, Fsa2PS automatically detects the applied dye set [6] and allocates the colours according to the analysed probes. The created PostScript depicts the chromatogram deduced from fragment analysis together with information on the analysis run above the displayed results (Figure 1(A)).

## Conclusion

Trace2PS and Fsa2PS provide easy to handle software toolkits implemented in Perl. Due to their platform independency, they can be integrated into a variety of software projects or data analysis pipelines. The PostScript output can afterwards be converted into various file formats like e.g. PDF, BMP, JPEG, GIF, PNG. Both tools are useful and applicable in data management workflow systems like SAMS (a sequence analysis and management system for ESTs; [7]), or laboratory information management systems (LIMS) of DNA core facilities that are used for displaying trace or fragment analysis results via web-based tools over an intranet or internet connection to users that can view their results directly on the screen.

## Competing interests

The authors declare that they have no competing interests.

## Availability and requirements

Toolkit names: Trace2PS, FSA2PS

Toolkits homepage: 

Operating system(s): Platform independent

Programming language: Perl

Other requirements: Perl 5.8 or higher

License: The software toolkits are open source and free for academic use.

## Authors' contributions

MW wrote the initial Trace2PS code according to a requirement profile from BW, and RN enhanced the code significantly. JK wrote the FSA2PS code, further enhanced the Trace2PS code. JK prepared the manuscript together with AG. All authors have read and approved the manuscript.
